# Structural characteristics and membrane interactions of tandem α-synuclein oligomers

**DOI:** 10.1038/s41598-018-25133-0

**Published:** 2018-04-30

**Authors:** Chunhua Dong, Marion Hoffmann, Xi Li, Meijing Wang, Craig R. Garen, Nils O. Petersen, Michael T. Woodside

**Affiliations:** 1 Department of Physics, University of Alberta, Edmonton, AB Canada; 2 Department of Chemistry, University of Alberta, Edmonton, AB Canada; 3 National Research Council, National Institute of Nanotechnology, University of Alberta, Edmonton, AB Canada

## Abstract

Pre-fibrillar oligomers of α-synuclein are thought to be pathogenic molecules leading to neurotoxicity associated with Parkinson’s disease and other neurodegenerative disorders. However, small oligomers are difficult to isolate for study. To gain better insight into the properties of small α-synuclein oligomers, we investigated engineered oligomers of specific size (dimers, tetramers, and octamers) linked head-to-tail in tandem, comparing the behavior of the oligomers to monomeric α-synuclein. All oligomeric constructs remained largely disordered in solution, as determined from dynamic light scattering and size-exclusion chromatography. Electron microscopy revealed that each construct could aggregate to form fibrils similar to those formed by monomeric α-synuclein. The interactions with large unilamellar vesicles (LUVs) composed of negatively-charged lipids differed depending on size, with smaller oligomers forming more extensive helical structure as determined by CD spectroscopy. Monitoring the influx of a fluorescence bleaching agent into vesicles showed that larger oligomers were somewhat more effective at degrading vesicular integrity and inducing membrane permeabilization.

## Introduction

Several lines of evidence have linked α-synuclein, an intrinsically disordered protein (IDP)^1^ that is enriched at the presynaptic termini of neurons^2^, with Parkinson’s disease (PD)^3^, a common neurodegenerative disorder typified by the loss dopaminergic neurons in the *substantia nigra* region of the brain^4^. The histopathological hallmark of PD is the intraneuronal accumulation of Lewy bodies^5,6^, cytosolic inclusions composed largely of β-structured amyloid fibrils of α-synuclein^7,8^. While PD is typically a sporadic condition, approximately 10% of cases are familial and can lead to early-onset forms of the disease. The root cause of heritable PD is either possession of one of several missense mutations in the *SNCA* gene encoding α-synuclein (A30P, E46K, H50Q, G51D, A53T or A53E) or gene multiplications of the wildtype allele^9–16^.

Soluble monomers of α-synuclein are known to aggregate into misfolded oligomers that lead to the fibrillar form^17^. Metastable pre-fibrillar oligomers, rather than the fibrils themselves, are thought to be the neurotoxic species implicated in PD ætiology^18–25^. The propensity of these pre-fibrillar molecules to assume amyloidogenic cross-beta sheet structure has been demonstrated^18,26^, as has their affinity for, and enrichment in, predominantly negatively-charged lipid membranes^27–36^. Although some studies have investigated the properties of intermediate-size oligomers of approximately 20–30 monomers^37,38^ and others have studied the formation of smaller oligomers^39–44^, there is still much to be learned about the properties of the smallest oligomers, in part because it is difficult to isolate them for study.

Studying oligomers is challenging on several fronts. It is unclear, for example, if methods for preparing oligomers *in vitro* consistently produce identically sized or structured oligomers^45–47^. Moreover, because oligomeric species are often transient, the conformations most relevant to neurotoxicity may be rare or relatively short-lived. They are also mixed in amongst the heterogeneous population of pre-fibrillar molecules transitioning along a misfolding/aggregation continuum from small oligomers to mature fibrils. Similar to some previous studies^48–54^, we have engineered small oligomers consisting of 2, 4, or 8 tandem repeats of α-synuclein connected head-to-tail by short peptide linkers, as model systems for studying the behavior of small oligomers and how it differs from that of monomers. This approach allows the protein to be studied under conditions where the local concentration of monomer units within the linked oligomer is high, inducing interactions that lead to the initiation of aggregation, while controlling the overall concentration of protein so that large aggregates are inhibited (at low concentration) or encouraged (at high concentration).

Here we have characterized the biophysical properties of each of these oligomeric constructs and compared them to monomeric α-synuclein. After purifying them to homogeneity^55^, we measured the hydrodynamic radius via dynamic light scattering and size-exclusion chromatography, as well as the secondary structure via circular dichroism (CD) spectroscopy, to look for changes in the structural properties as a function of oligomer size. We also verified their ability to form amyloid fibrils. Finally, we examined their interactions with lipid bilayers and vesicles, using CD spectroscopy to monitor any lipid-induced conformational changes in each construct and a fluorescence bleaching assay to monitor disruption of the membrane induced by the constructs.

## Results

### Size characterization of tandem oligomers

**Figure 1:**
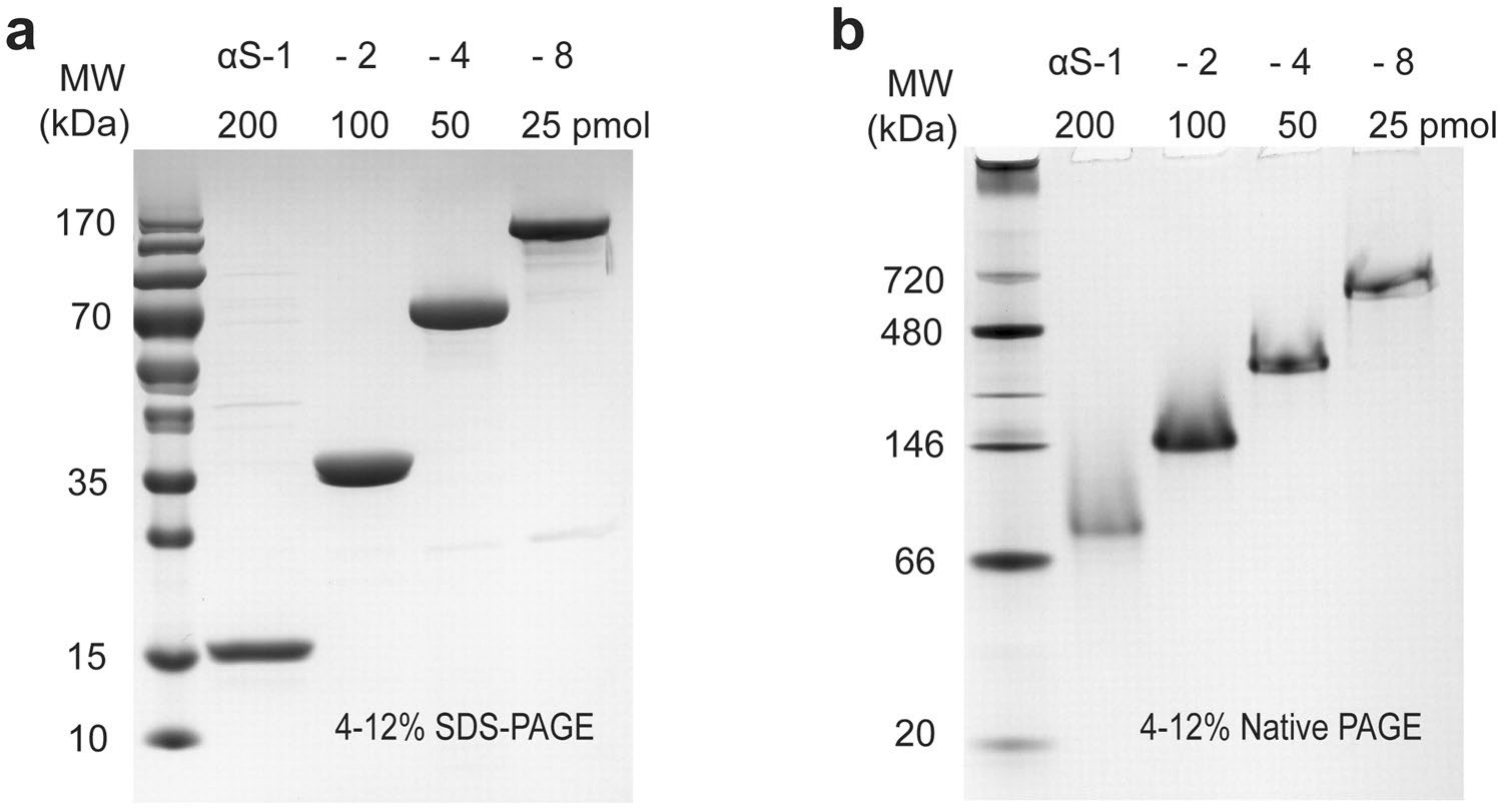
Polyacrylamide gel analysis of α-synuclein constructs. (**a**) SDS-PAGE gradient (4–12%) gel analysis of purified αS-1, αS-2, αS-4, and αS-8 recombinant proteins. Each lane was loaded with protein as indicated to give equivalent amounts of monomer. (**b**) Native PAGE analysis of recombinant proteins. The band for each α-synuclein construct runs above the molecular weight expected.

Recombinant proteins consisting of monomeric α-synuclein (αS-1), tandem dimers (αS-2) formed from monomers linked by a tri-peptide, tandem tetramers (αS-4) consisting of dimers linked by a tri-peptide, and tandem octamers (αS-8) formed from tetramers linked by a tri-peptide were purified in yields of 10–20 mg per litre of expression culture for monomers, dimers, and tetramers, and in yields of 1–2 mg/L for octamers. Following purification, protein preparations were shown to be of high purity using SDS- and native-PAGE analysis (Fig. 1). The molecular mass of each construct calculated using the ProtParam tool, respectively 14,460, 29,103, 58,390, and 116,963 Da for αS-1 through αS-8, agreed well with SDS-PAGE analysis (Fig. 1a) and the mass measured by mass spectrometry: respectively 14,464, 29,104, 58,390, and 116,972 Da. Native PAGE analysis, however, revealed a single band for each recombinant protein at an apparent molecular weight much larger than its actual mass when compared to a standard protein ladder (Fig. 1b). The higher-than-expected apparent molecular weight, combined with the lack of evidence for higher-order aggregates, is consistent with the notion that the constructs are extended but largely disordered.

**Figure 2:**
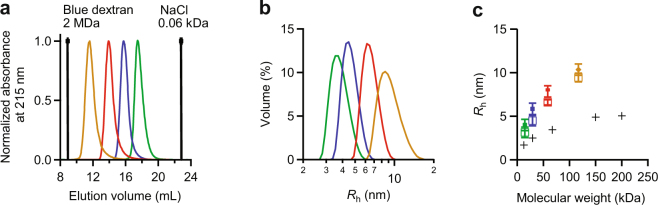
Hydrodynamic radius analysis from SEC and DLS. (**a**) SEC analysis showing the normalized elution peaks for individual runs of αS-1 (green), αS-2 (blue), αS-4 (red), and αS-8 (yellow). (**b**) Volume-averaged size distribution curves from triplicate DLS measurements of αS-1 (green), αS-2 (blue), αS-4 (red), and αS-8 (yellow). (**c**) Comparison of *R*_h_ determined from SEC (circles) and DLS (squares) for αS-1 (green), αS-2 (blue), αS-4 (red), and αS-8 (yellow) (error bars: s.e.m.). The results are comparable: *R*_h_ increases sublinearly with molecular mass. Black crosses: results for standard proteins of known molecular weight and *R*_h_.

We estimated the hydrodynamic radius of each construct using size exclusion chromatography (SEC), with a column calibrated against protein standards having a range of molecular weights similar to those of the constructs. Each α-synuclein construct eluted as a single peak earlier than the protein standard of corresponding weight (Fig. 2a), again showing that the constructs are all extended in conformation. These results were confirmed by dynamic light scattering (DLS) measurements. We first measured protein standards to generate a standard curve for the hydrodynamic radius, *R*_h_, then we determined *R*_h_ for each α-synuclein construct in turn (Fig. 2b). The α-synuclein constructs had distinctly larger *R*_h_ than globular proteins of similar molecular mass. The results for *R*_h_ from SEC and DLS were consistent within experimental uncertainty (Fig. 2c). Because both the native gel and SEC show that higher-order aggregates did not form under the conditions of these measurements, the larger-than-expected *R*_h_ values in Fig. 2c indicate extended conformations for all of the α-synuclein constructs.

### Fibrillization of tandem oligomers

**Figure 3:**
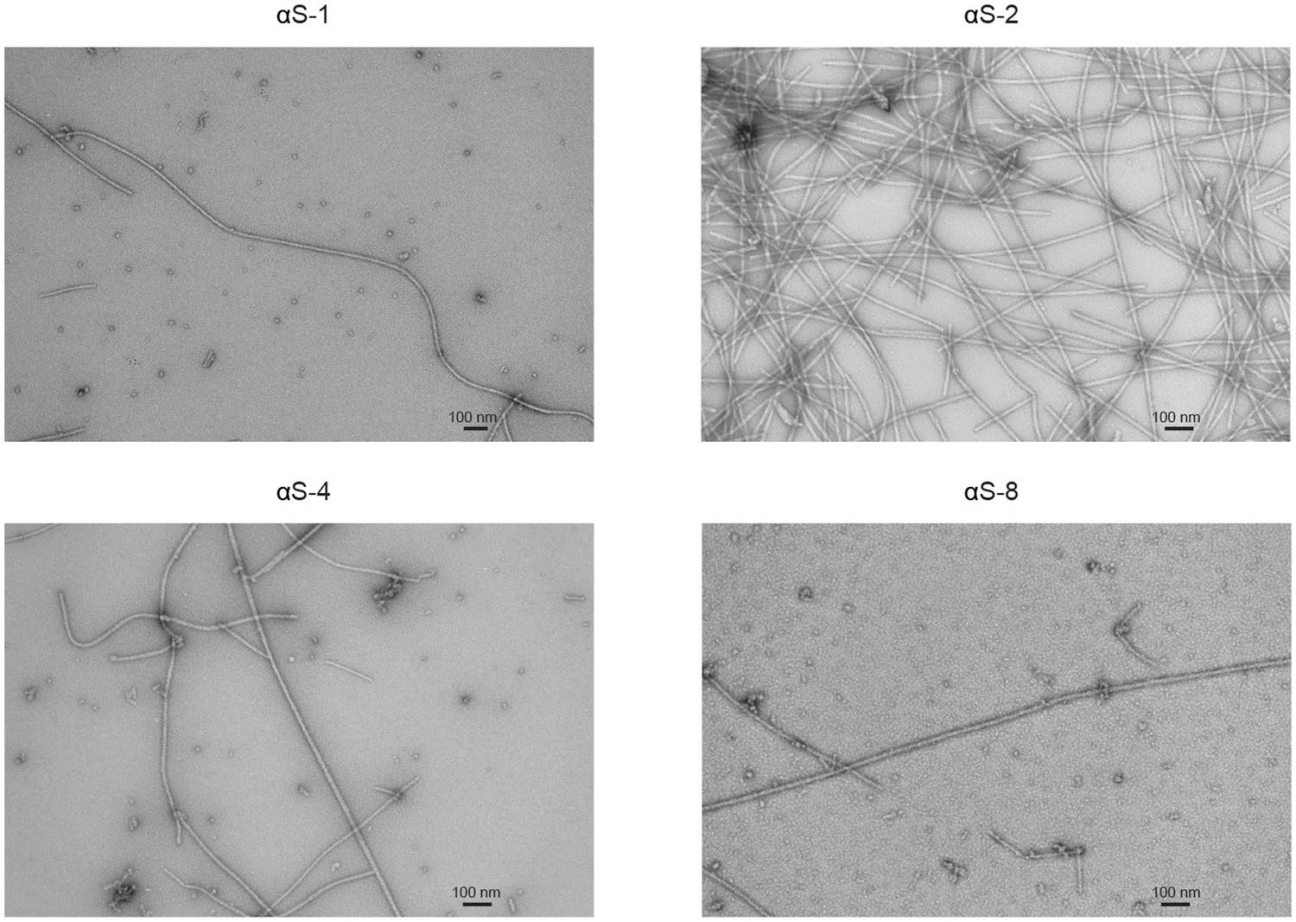
Electron microscopy of fibrils. TEM micrographs of fibrils formed by α-synuclein constructs show that each form morphologically similar fibrils. Scale bars: 100 nm. Fibrils were formed under similar but not identical conditions: 2 mg/mL monomer in 400 μL volume, 2.2 mg/mL dimer in 300 μL volume, 1.1 mg/mL tetramer in 300 μL volume, and 1.0 m/mL octamer in 150 μL volume.

The ability of each construct to form fibrils was tested by incubating each protein at 37 °C with shaking, and then examining the result in an electron microscope. In each case, long amyloid fibrils were observed (Fig. 3). Comparing the fibrils formed by the tandem oligomers to those formed by wild-type monomers under direct magnification of 25,000–30,000x, we found that they all had qualitatively similar widths. The apparent fibril width was ~12–15 nm at the widest point of a single fibril; at the cross-over in the image of αS-8 fibrils, it was 14–21 nm. The similarity between the fibrils formed by each construct suggests that the monomer units all end up packed into very similar conformations in mature fibrils, despite the constraint of the peptide linker between tandem repeats.

**Figure 4:**
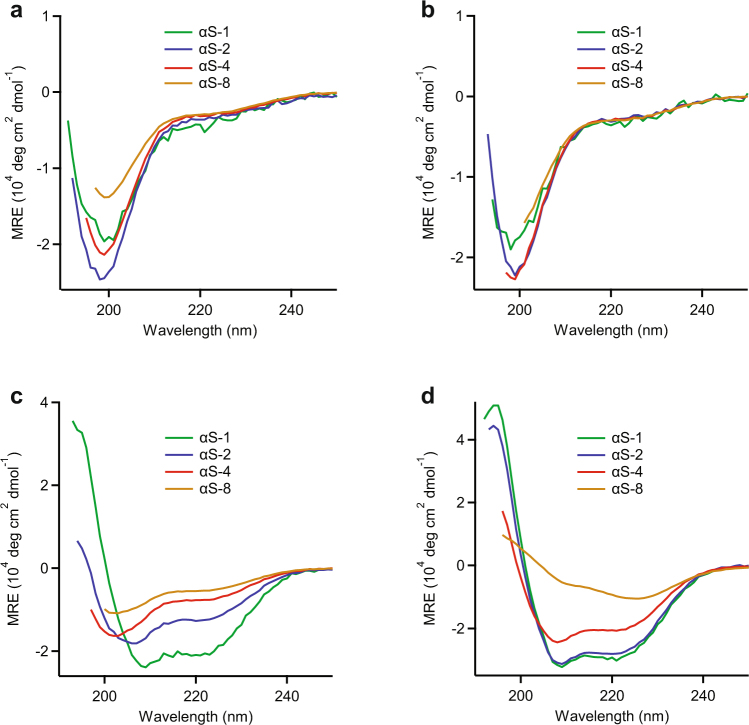
CD spectra of α-synuclein species incubated with vesicles of different composition. (**a**) The CD spectra of all α-synuclein constructs in PBS buffer are characteristic of unfolded, random coils. (**b**) The CD spectra of all α-synuclein constructs are unchanged when incubated with LUVs composed of POPC. (**c**) CD spectra for α-synuclein constructs incubated with LUVs composed with POPS reveal conversion to varying amounts of helical structure. (**d**) CD spectra of α-synuclein constructs incubated with LUVs composed of SOPG show marked α-helical structure formation for αS-1, αS-2, and αS-4, but different behavior for αS-8.

CD spectroscopy of structural changes upon lipid bilayer binding. CD spectroscopy was used to monitor the secondary structure of the different α-synuclein constructs, both alone in phosphate-buffered saline (PBS) at pH 7.4, and in the presence of various phospholipid bilayer vesicles. In PBS alone (Fig. 4a), all three tandem-oligomer constructs showed CD spectra that were effectively the same as the spectrum for monomeric α-synuclein, indicating that all of the tandem oligomers were similarly disordered as the monomer. This result was consistent with the picture indicated by the *R*_h_ values. In the presence of 1-palmitoyl-2-oleoyl-*sn*-glycero-3-phosphocholine (POPC), an uncharged phospholipid, the results were almost unchanged, suggesting that either the α-synuclein constructs did not interact with the uncharged POPC LUV, or else they formed no detectable secondary structure if they did interact (Fig. 4b). When vesicles of 1-palmitoyl-2-oleoyl-*sn*-glycero-3-phospho-L-serine (POPS), an anionic phospholipid, were mixed with the proteins instead, the CD spectra of both the monomer and dimer displayed features characteristic of α-helical secondary structure (a double peak in absorption at 222 and 208 nm), with the helical character most pronounced in the monomer (Fig. 4c). In contrast, the spectra of the tetramer and octamer were little changed from the addition of the POPS vesicles, indicating that these proteins remained unstructured. The most dramatic changes were seen when vesicles of another anionic phospholipid, (SOPG) were added to solutions of the different constructs (Fig. 4d): the monomer and dimer both took on very high levels of helical structure, the tetramer displayed distinct helical content, and the spectrum of the octamer shifted consistent with the gain of some modest alpha-helical character. Such structural rearrangements induced by interactions with a negatively charged membrane matches previous results^27,42,56^. However, we see a consistent pattern whereby the amount of helical structure decreases as the size of the oligomer increases, indicating that the larger oligomers are less prone to structural remodeling upon membrane binding.

### Permeabilization of lipid bilayers

**Figure 5:**
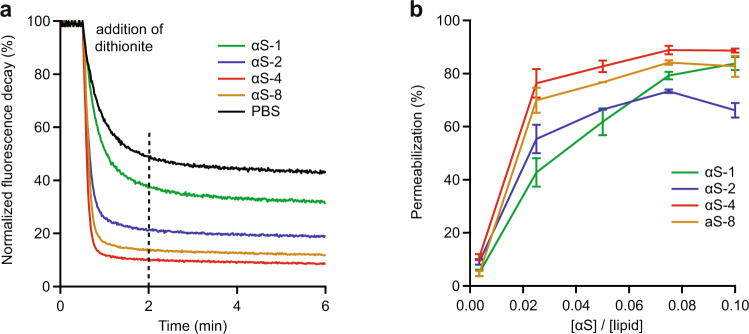
Permeabilization of SOPG LUVs by α-synuclein oligomers. (**a**) Fluorescently labelled LUVs premixed with oligomers were mixed with dithionite, causing a decrease of fluorescence as the dye was bleached by the dithionite (αS-1, green; αS-2, blue; αS-4, red; αS-8, yellow; PBS, black). Without α-synuclein present, only the outer leaflet was bleached; additional bleaching occurred in the presence α-synuclein of owing to bleaching of the inner leaflet after membrane permeabilization. (**b**) The percentage of membrane permeabilization 2 min after dithionite addition was calculated for each construct at different concentrations of α-synuclein and plotted as a function of the molar ratio of protein to lipid.

One of the mechanisms proposed for α-synuclein toxicity is that it permeabilizes the cell membrane^57,58^. We therefore tested the relative ability of each tandem oligomer construct to permeabilize membranes, compared to the wild-type monomer, using LUVs with membranes containing a mixture of SOPG and fluorescently labelled C12-NBD-PG. Vesicles were incubated with protein at various concentrations, then sodium dithionite was added to bleach the C12-NBD-PG molecules. Only the outer leaflet of the vesicle would be bleached if the vesicle remained intact, leaving the fluorescence of the inner leaflet untouched, whereas disruption of the membrane by α-synuclein would lead to bleaching of the inner leaflet, too^38^. NBD fluorescence was measured as a function of time (Fig. 5a) following addition of reductant to solutions of pre-incubated LUVs and α-synuclein constructs. Without any α-synuclein present (Fig. 5a, black), the fluorescence decayed rapidly to roughly half of its initial value as the outer leaflet was bleached, followed by a much slower decay arising from the bleaching of C12-NBD-PG molecules^59^ that slowly transferred from the inner leaflet to the outer leaflet^60^. With α-synuclein present (Fig. 5a, green, blue, red and yellow), the initial fluorescence decay was larger because some of the vesicles were permeabilized, leading to the bleaching of both inner and outer leaflets.

To quantify the extent of permeabilization, we measured the magnitude of the fluorescence drop 2 min after the introduction of sodium dithionite, as a function of the protein:lipid molar ratio for each construct αS-1 through αS-8 (Fig. 5b). At the lowest molar ratio (1:300), similarly low permeabilization (~5–10%) was observed for all constructs. At intermediate concentrations, the tetramer (Fig. 5B, red) and octamer (Fig. 5b, yellow) were noticeably more effective at permeabilizing the membranes than the monomer (Fig. 5b, green) and dimer (Fig. 5b, blue). At higher concentrations, the effectiveness of the dimer, tetramer, and octamer did not improve much, but that of the monomer did, such that at the highest concentration (7 μM), the monomer was similar to the tetramer and octamer.

## Discussion

The results from the structural probes indicate that each of the oligomers studied here remained largely disordered like the monomer, rather than developing a more compact conformation as found previously in larger oligomers^38,61–63^ or the proposed native helical tetramer^39^. The implication is that no major structural reorganization occurred when going from monomers to octamers. Previous single-molecule force spectroscopy studies found that these tandem oligomers can, occasionally, form transient structures^53,54^, but on average the behavior was still disordered, consistent with the ensemble results reported here. Intriguingly, studies of oligomer growth using single-molecule fluorescence found that oligomers started to undergo a structural reorganisation once they grew to be around 8–10 monomers or more in size^41^, close to the largest oligomer we studied. We did not observe any obvious sign of such changes, however, possibly because the ensemble probes we used are less sensitive than single-molecule methods and the changes were too small to be detected.

Turning to the fibrils formed by the tandem-repeat oligomers, the fact that the transmission electron micrographs showed morphologically similar fibrils for all of the constructs studied indicates that each of the constructs was able to achieve the same final end-point in the aggregation process, producing amyloid fibril like the wildtype monomer. The width of the fibrils formed by each tandem-repeat oligomer was also consistent with the range of fibril widths reported previously for α-synuclein (approximately 10–20 nm)^64–66^. These results suggest that linking the monomer units together in tandem does not significantly constrain the conformational space explored by each monomer unit, allowing them to form standard amyloid structures. Furthermore, the similar morphology for fibrils formed by wild-type monomers compared to fibrils formed by tandem-repeat oligomers consisting of monomers units arranged head-to-tail suggests that a head-to-tail orientation of individual monomers may represent at least one of the conformers relevant for fibrillization^63^.

**Figure 6:**
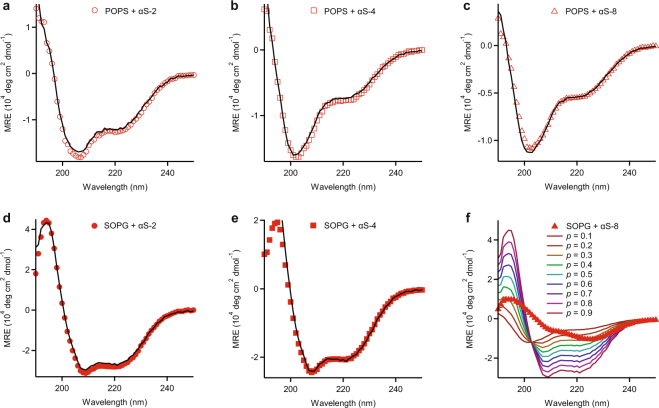
Fitting of CD spectra for tandem-repeat oligomers with anionic phospholipid vesicles. The CD spectra (red symbols) for (**a**) αS-2 and POPS, (**b**) αS-4 and POPS, (**c**) αS-8 and POPS, (**d**) αS-2 and SOPG, and (**e**) αS-4 and SOPG were fit to a linear combination of the spectrum for helical and disordered αS-1 (black line). (**f**) The CD spectrum for αS-8 and SOPG (solid red triangles) could not be fit to any linear combination of helical and disordered monomer spectra (coloured lines at different fractions *p* of helical structure).

Differences in the behavior of different oligomers were seen primarily in the interactions with vesicles. Whereas all oligomers remained disordered in the presence of vesicles composed of uncharged lipids and underwent some form of structural reorganization in the presence of vesicle made of negatively charged lipids, as seen previously for monomeric α-synuclein^27,31,66^, the CD spectra suggest that the nature of the structural reorganization was somewhat different for the different oligomers. The spectra of the α-synuclein constructs with POPS (Fig. 4c) appear to show a gradual change from high helical content for monomers to low helical content and considerable disorder in octamers. Hypothesizing that these spectra can be separated into two components, one helical (like the monomer) and the other disordered, we fit the spectra for the different oligomers to a linear combination of the spectra for αS-1 in POPS (helical) and αS-1 in PBS (disordered): *p* × MRE_αS-1/POPS_ + (1–*p*) × MRE_αS-1/PBS_. We found that *p* varied inversely with the number of monomer units in the oligomer, *i.e*. *p* = 0.51 ± 0.01 for αS-2, 0.21 ± 0.01 for αS-4, and 0.16 ± 0.01 for αS-8, indicating that likely only one of the monomer subunits in each oligomer changed to a helical conformation upon interaction with the vesicle, with the others remaining disordered (Fig. 6a–c). However, the same was not true for SOPG: fitting the equivalent two components gave *p* = 0.89 ± 0.01 for αS-2 and 0.67 ± 0.01 for αS-4 (Fig. 6d,e), indicating that most of the tetramer and almost all of the dimer became structured like the monomer, but the octamer spectrum could not be decomposed in this way (Fig. 6f), indicating that it formed a structure that was distinct from the helical conformation of the vesicle-bound monomer. The differences between the effects of POPS and SOPG may be related to the observation that oligomer insertion into a bilayer depends not only upon the phospholipid net charge but also the relative ease of access to the hydrophobic core^47^, since SOPG and POPS have different chain lengths and head groups.

With respect to the permeabilization of SOPG vesicles, only minor differences between the different α-synuclein constructs were seen. Each of the oligomers was able to induce vesicle permeabilization on a similar time-scale as seen for monomeric α-synuclein. Given that permeabilization is hypothesized to occur via formation of oligomeric α-synuclein pores having largely helical structure^23,67–73^, it is very intriguing that the octamer can induce permeabilization just as effectively as the other constructs, despite having notably less helical content. Possible explanations include the notions that only part of each octamer contributes to the helical pore-forming structure, that some fraction of octamers form helical pores but the rest reconfigure into a different structure, or that the architecture of the pore formed by the octamers is distinct from the pores formed by the other constructs. Finally, we note that the concentration dependence of the permeabilization seen in Fig. 5b suggests a saturation effect at high protein concentration, possibly reflecting reports from studies modelling membrane disruption that found α-synuclein insertion can impair lipid diffusion^74^, thereby preventing efficient pore formation at high concentration. Similar concentration dependence and saturation effects have been seen previously in anionic model vesicle permeabilization assays using both monomeric and oligomeric α-synuclein at similar concentrations to those we used^47,75,76^.

In summary, this work characterizing the properties of small tandem-repeat oligomers of α-synuclein finds that they recapitulate much of the behavior of monomeric α-synuclein in terms of their generally disordered structure, the fibrils they form, and their ability to interact with vesicles and permeabilize them, although small differences were seen in the structures the oligomers formed in the presence of vesicles. These results suggest that the end-to-end linkage of monomers in the tandem repeats does not significantly change the behavior of the protein.

## Methods

### Design and cloning of α-synuclein tandem-oligomer constructs

Tandem repeat constructs of α-synuclein were designed by linking the C-terminus of one full-length α-synuclein monomer to the N-terminus of the next using a short, flexible, glycine-serine-glycine (GSG) linker. Using this design scheme, we generated αS-2, αS-4, and αS-8 constructs from the open reading frame encoding αS-4, synthesized and cloned into a pJexpress-406 expression vector (DNA2.0). To obtain αS-2, the central dimer from the αS-4 expression vector was excised via BamHI restriction endonuclease sites engineered into the sequence, and then re-ligated into the vector for expression. αS-8 was generated by first making a new copy of the αS-4 insert using PCR amplification, then digesting the PCR product with NdeI and Csp61 (Thermo Fisher Canada, Mississauga, ON) and ligating it into αS-4 expression vector that had been previously linearized by NdeI digestion and dephosphorylated with calf-intestinal alkaline phosphatase (Thermo Fisher Canada, Mississauga, ON), using the TaKaRa ligation kit for long fragments (Clontech, Mountain View, CA, USA). To increase expression yields, each coding sequence was subsequently sub-cloned from pJexpress-406 vectors into pET21a expression vectors (EMD Millipore, Etobicoke, ON, Canada).

### Expression and purification of α-synuclein tandem-oligomer constructs

Recombinant proteins were over-expressed by transforming Rosetta 2 (DE3) competent cells (EMD Millipore, Etobicoke, ON, Canada) to ampicillin resistance using pET21a expression plasmids containing the respective coding sequences for each tandem-oligomer construct. 1 L LB medium supplemented with 100 μg/mL ampicillin and 34 μg/mL chloramphenicol (SigmaAldrich, Oakville, ON, Canada) was inoculated to an OD_600 nm_ of 0.1 with an overnight culture, and then incubated at 37 °C at 225 rpm until an OD_600 nm_ of 0.6 was reached. Protein over-expression was induced by adding isopropyl β-D-1-thiogalactopyranoside (IPTG; GoldBio, St. Louis, MO, USA) to a final concentration of 2 mM, then cells were further cultivated at 30 °C and 225 rpm for 5 hours before harvesting by centrifugation.

All α-synuclein constructs were purified without using affinity purification tags via an osmotic shock protocol described previously^55^, with minor modifications and the addition of fractionated ammonium sulfate precipitation steps. Cells from 1 L of expression culture were re-suspended in 50 mL osmotic shock buffer (30 mM Tris-Cl pH 7.2 at room temperature, 40% (w/v) sucrose, 2 mM EDTA) and incubated at room temperature for 10 min. Cells were then pelleted by centrifugation at 21,000 *g* for 40 min and the pellet was re-suspended in 90 mL cold MilliQ H_2_O supplemented with 37.5 μL of saturated MgCl_2_. The sample was kept on ice for 10 min and then centrifuged at 21,000 *g* for 40 min to pellet the cells. Following centrifugation, the supernatant containing proteins released from the periplasm of the *E. coli* cells was subjected to ammonium sulfate precipitation. The solution was gently stirred in a glass beaker and a 90% saturated ammonium sulfate (AS) solution was added. Monomers were precipitated with 40% AS, whereas all oligomers were precipitated at a concentration of 35% AS. Proteins were allowed to precipitate fully over night at 4 °C with gentle stirring, then were pelleted by centrifugation at 75,600 *g* for 60 min. The supernatant was decanted and the pellets were further purified by anion exchange chromatography.

Pellets from 1 L of culture volume were dissolved in 90 mL of Q-Sepharose buffer A (20 mM Tris-Cl pH 8.0) and applied to an equilibrated 30 mL Q-Sepharose FF (GE Life Sciences, Mississauga, ON, Canada) column using an AKTA Purifier 100 FPLC system (GE Life Sciences, Mississauga, ON, Canada). Unbound proteins were washed off with 10 column volumes of buffer A and then the α-synuclein was eluted from the column using a gradient of 0–50% of buffer B (20 mM Tris/HCl, 1 M NaCl, pH 8.0). Fractions were analyzed by SDS-PAGE; those containing α-synuclein were pooled and the protein was precipitated again with ammonium sulfate as described above. At this point protein pellets were either frozen in liquid nitrogen for long term storage at −80 °C or purified in a final step by size exclusion chromatography (SEC).

For SEC, ammonium sulfate pellets were dissolved in cold phosphate buffered saline (PBS; 137 mM NaCl, 2.7 mM KCl, 10 mM Na_2_HPO_4_, 2 mM KH_2_PO_4_, pH 7.4) to yield a protein concentration <3 mg/mL. For αS-1, -2, and -4; a Sephacryl S200 HiPrep 26/60 column (GE Life Sciences, Mississauga, ON, Canada) with a separation range of 5,000–250,000 Da was used, whereas for αS-8, a Sephacryl S300 HiPrep 26/60 column (GE Life Sciences, Mississauga, ON, Canada) with a separation rage of 10,000–1,500,000 Da was used. SEC fractions were analyzed on a SDS-PAGE gel and the pure fractions were pooled. In most cases, SEC-purified protein was used immediately for all further experiments without freezing aliquots. The purity of all constructs was > 95% as determined by colloidal Coomassie-stained SDS-PAGE. Protein concentrations were determined spectrophotometrically using Beer’s law after measuring the absorbance at 280 nm with a Cary 60 UV/Vis spectrophotometer (Agilent Technologies, Mississauga, ON, Canada) and using theoretical extinction coefficients of 5960 M^−1^ cm^−1^, 11920 M^−1^ cm^−1^, 23840 M^−1^ cm^−1^, and 47680 M^−1^ cm^−1^ for each of αS-1, αS-2, αS-4, and αS-8, respectively (ProtParam, ExPASy).

### Polyacrylamide gel electrophoresis (PAGE)

Yield and purity of αS-1 was analyzed with 15% SDS-PAGE, αS-2 and αS-4 with 12% SDS-PAGE, and αS-8 on 8% SDS-PAGE continuous gels using standard buffers^77^. All proteins were analyzed together using 4–12% gradient Novex Tris-Glycine gels (Thermo Fisher Canada, Mississauga, ON). Native gels were run with the Novex Tris-Glycine Native Running Buffer and Novex Tris-Glycine Native Sample Buffer using the NativeMark Unstained Protein Standards (Thermo Fisher Canada, Mississauga, ON) as molecular mass markers. SDS gels were run with Novex Tris-Glycine SDS Running Buffer and Novex Tris-Glycine SDS Sample Buffer and the Fermentas 10–170 kDa PageRuler Prestained Protein Ladder (Thermo Fisher Canada, Mississauga, ON). Novex gels were run at 125 V and all gels were stained with PageBlue protein staining solution (Thermo Fisher Canada, Mississauga, ON).

### Analytical SEC

A set of standard proteins of known molecular mass and hydrodynamic radius (*R*_h_) was re-suspended according to the manufacturer’s recommendations (SigmaAldrich, Oakville, ON, Canada) in PBS pH 7.4. Each standard was run in triplicate to determine its elution volume (*V*_e_) from a Superose 6 10/300 GL analytical size exclusion chromatography column (GE Life Sciences, Mississauga, ON, Canada) with separation range between 5 kDa to 5 MDa. The injection volume was 240 μL, the flow-rate 0.5 mL/min, the temperature 4 °C, and PBS pH 7.4 was used as buffer. A calibration curve was generated by plotting 1000/*V*_e_ versus *R*_h_ as described^78^. α-Synuclein monomer and tandem-oligomer constructs were applied to the column at concentrations ranging between 0.5 and 2.5 mg/mL and their *R*_h_ values determined from the calibration curve.

For analytical SEC under denaturing conditions, protein constructs were first precipitated with ammonium sulfate. The protein pellets were then dissolved to approximately 1 mg/mL in the unfolding buffers and incubated at room temperature for at least one hour before being applied to the column. Denaturation buffers were 8 M urea or 6 M guanidinium HCl in PBS buffer.

### Dynamic light scattering

Dynamic light scattering (DLS) experiments were performed at 25 °C using a Zetasizer Nano ZS instrument (Malvern Instruments Ltd., Malvern, UK) following the manufacturer’s instructions. The excitation wavelength of the laser was 633 nm, and backscattered light was detected at an angle of 173°. PBS pH 7.4 was used as dispersant for all protein samples and the manufacturer-recommended standard operating procedures and settings were used: respectively 1.45 and 0.001 for the refractive index and absorption of the protein, and respectively 1.334 and 0.911 cP for the refractive index and viscosity of PBS as dispersant. Duration time and attenuation were set to automatic, the sample was equilibrated for 120 s before starting the measurements, 3 measurements were performed for each sample, and an extended duration was allowed for large particles.

DLS measurements were also used to determine *R*_h_ for various reference proteins from the Gel Filtration Markers Kits for Protein Molecular Weights 12,000–200,000 Da and 29,000–700,000 Da (SigmaAldrich, Oakville, ON, Canada). Samples were measured at concentrations ranging from 0.5 to 4 mg/mL after filtration into the cuvettes (Hellma 105.251-QS, d = 0.3 cm; Hellma Canada Ltd, Markham, ON) using Anotop 0.1 µm syringe filters (Whatman; Thermo Fisher Canada, Mississauga, ON). Cuvettes were sealed, measured, and the results were compared to those of the α-synuclein constructs.

### Preparation of protein fibrils for transmission electron microscopy

Fibrils of all α-synuclein constructs grew within several days to weeks by incubating the proteins in PBS at 37 °C, shaking at 300 rpm. Fibrils were prepared from monomers, dimers, and tetramers at concentrations respectively of 2.0, 2.2, and 1.1 mg/mL in volumes of 300–400 µL, using microcentrifuge tubes with screw caps and O-rings to avoid evaporation incubated in a Fisher Scientific thermal mixer (Thermo Fisher Canada, Mississauga, ON). Because lower amounts of the octamer were available, it was fibrillized in wells of a sealed 96-well plate (Greiner Bio-One, Monroe, NC, USA), with a siliconized glass bead (3 mm) added to 150 µL of 1.0-mg/mL protein solution in each well. A plate reader was used to control the temperature and shaking. For transmission electron microscopy, fibril samples were diluted (αS-1 and αS-2: to 0.5 mg/mL, αS-4: to 0.55 mg/mL, and αS-8: to 0.5 mg/mL) and then spotted onto EM grids and stained with 1% uranyl acetate. Micrographs were collected using an accelerating voltage of 80 kV. The direct magnification was 25000 × (αS-1) or 30000 × (other constructs). Fibril widths were estimated qualitatively by direct measurement from the micrographs.

### Preparation of large unilamellar vesicles (LUVs)

The phospholipids 1-palmitoyl-2-oleoyl-*sn*-glycero-3-phosphocholine (sodium salt) (POPC; Avanti Polar Lipids, Alabaster, AL, USA), 1-palmitoyl-2-oleoyl-*sn*-glycero-3-phospho-L-serine (sodium salt) (POPS; Avanti Polar Lipids, Alabaster, AL, USA), and 1-stearoyl-2-oleoyl-*sn*-glycero-3-phospho-(1′-*rac*-glycerol) (sodium salt) (SOPG; Avanti Polar Lipids, Alabaster, AL, USA) were obtained as powders and stored at −20 °C until use. PBS buffer at was added to the phospholipids in glass vials to obtain a lipid concentration of 10 mM, and the solution was shaken at 500 rpm for 1 hour at room temperature. Afterwards the sample was vortexed vigorously, then the solution was passed eleven times through two 100-nm pore-size polycarbonate membranes laid atop one another using a Mini-Extruder device (Avanti Polar Lipids, Alabaster, AL, USA). The LUVs generated by this method were stored at 4 °C and checked before use by DLS for a uniform size distribution of approximately 100 nm. Large unilamellar vesicles (LUVs) were measured in disposable DTS0012 cuvettes (Malvern Instruments, Inc., Malvern, UK) by pipetting 1.5 mL of sample solution into the cuvette. Settings were 1.33 and 0.8882 cP for the dispersant refractive index and viscosity, respectively, and 1.43 and 0.01 for the phospholipid refractive index and absorption, respectively.

Fluorescent LUVs were prepared in HPLC-grade chloroform by mixing 10 mM SOPG with 100 µM of the fluorescently labeled phospholipid 1-palmitoyl-2-{12-[(7-nitro-2-1,3-benzoxadiazol-4-yl)amino]dodecanoyl}-*sn*-glycero-3-[phospho-*rac*-(1-glycerol)], (C12-NBD-PG; Avanti Polar Lipids, Alabaster, AL, USA). The organic solvent was removed by drying using a gentle stream of nitrogen to yield a film of the lipid mixture, which was further dried under vacuum for 24 h using a Freezone 2.5 Plus lyophilizer (Labconco, Inc., Kansas City, MO, USA) to remove any residual solvent. The lipid film was then hydrated using 10 mM HEPES, 0.9% w/v NaCl, pH 7.4, and the suspension was incubated for 1 hour at 20 °C while shaking at 400 rpm. LUVs of a diameter of approximately 100 nm were produced as above. The mean fluorescent vesicle diameter was determined for individual LUV preparations from triplicate measurements using DLS, with settings identical to those used above for non-labelled LUVs. From the mean diameter *d* = 1.2 ± 0.2 × 10^2^ nm, we approximated the number of phospholipids present per unilamellar vesicle (*N*_tot_) via:

1 $${N}_{tot}=(4\pi /a)[{(d/2)}^{2}+{(d/2-h)}^{2}],$$

where *a* is the lipid head group area, approximately 0.71 nm^2^, and *h* is the bilayer thickness, 5 nm^79^. From the result, 1.1 × 10^5^, we deduced a LUV molar concentration of 0.9 nM.

### CD spectroscopy

CD spectra were collected between 190 and 250 nm with a JASCO J–810 CD/ORD spectrometer (JASCO, Easton, MD, USA), using Starna quartz cuvettes (Starna Cells, Inc., Atascadero, CA, USA) with a path length of 0.01 cm, while scanning at a speed of 10 nm/min with a 4-s response time. The spectrum of each construct was measured at least 3 times at 3 different concentrations (1, 0.75, and 0.5 mg/mL) in PBS, averaging the results from all measurements. Spectra were baseline-corrected with the appropriate buffer spectrum. The mean residue molar ellipticity (MRE) was averaged from all spectra for a given construct. Because of high absorption below 195 nm in the high-concentration samples, results below 195 nm were inaccurate for the monomer and dimer and not included in the analysis; for the tetramer and octamer, the cut-off was 197–200 nm.

To investigate binding of ΑS tandem constructs to LUVs, 15 uM protein was mixed with 4.5 mM of either POPC, POPS or SOPG phospholipids (molar ratio protein:phospholipids 1:300, sufficiently low to ensure minimal disruption of the membrane by the protein) and incubated at room temperature in PBS. Spectra were measured after a 3-hour incubation using the settings described above. MREs from triplicate measurements were calculated and corrected with the baseline spectra of samples containing 4.5 mM of each LUV in PBS.

### Fluorescent LUV permeabilisation assay

Fluorescently labeled SOPG/C12-NBD-PG LUVs in PBS were incubated at room temperature with individual α-synuclein constructs for 1 hr. Permeabilization of the LUV membranes was monitored by adding reductant to the buffer to bleach the fluorescence of solvent-exposed C12-NBD-PG. C12-NBD-PG fluorescence was measured using a Photon Technology International QuantaMaster400 spectrofluorometer (HORIBA Scientific (Canada), London, ON) at an excitation wavelength of 466 nm, quantifying the fluorescence emission at 535 nm (2-nm bandwidths). Bleaching was initiated by adding 1 M sodium dithionite to the buffer, and the fluorescence was measured over the next 2 min. Measurements were repeated with different protein construct concentrations (0.3–7 µM for each construct) to achieve protein:lipid molar ratios ranging from 1:300 to 1:10, and compared to a positive control in which LUVs were lysed by 0.1% (v/v) Triton X-100 (SigmaAldrich, Oakville, ON, Canada).

To determine the total extent of membrane leakage, the fluorescence intensity was measured 2 min after dithionite addition. The fractional amount of permeabilization was calculated as $|{I}_{\alpha S}-{I}_{PBS}|/{I}_{PBS}$, where *I*_αS_ is the fluorescence intensity 2 min after adding dithionite to vesicles incubating with α-synuclein and *I*_PBS_ is the fluorescence intensity 2 min after adding dithionite to vesicles in PBS without any α-synuclein present.

### Data availability

All data are available from the corresponding author upon reasonable request.
